# Functional photoacoustic imaging: from nano- and micro- to macro-scale

**DOI:** 10.1186/s40580-023-00377-3

**Published:** 2023-06-19

**Authors:** Byullee Park, Donghyeon Oh, Jeesu Kim, Chulhong Kim

**Affiliations:** 1 Departments of Convergence IT Engineering, Mechanical Engineering, and Electrical Engineering, School of Interdisciplinary Bioscience and Bioengineering, Medical Device Innovation Center, Pohang University of Science and Technology (POSTECH), Pohang, 37673 Republic of Korea; 2 Caltech Optical Imaging Laboratory, Andrew and Peggy Cherng Department of Medical Engineering, California Institute of Technology, Pasadena, CA 91125 USA; 3 Department of Biophysics, Institute of Quantum Biophysics, Sungkyunkwan University, Suwon, 16419 Republic of Korea; 4 Departments of Cogno-Mechatronics Engineering and Optics and Mechatronics Engineering, College of Nanoscience and Nanotechnology, Pusan National University, Busan, 46241 Republic of Korea

**Keywords:** Biomedical imaging, Photoacoustic imaging, Ultrasound imaging, Functional photoacoustic imaging, Multiscale photoacoustic imaging

## Abstract

Functional photoacoustic imaging is a promising biological imaging technique that offers such unique benefits as scalable resolution and imaging depth, as well as the ability to provide functional information. At nanoscale, photoacoustic imaging has provided super-resolution images of the surface light absorption characteristics of materials and of single organelles in cells. At the microscopic and macroscopic scales. photoacoustic imaging techniques have precisely measured and quantified various physiological parameters, such as oxygen saturation, vessel morphology, blood flow, and the metabolic rate of oxygen, in both human and animal subjects. This comprehensive review provides an overview of functional photoacoustic imaging across multiple scales, from nano to macro, and highlights recent advances in technology developments and applications. Finally, the review surveys the future prospects of functional photoacoustic imaging in the biomedical field.

## Introduction

In the biomedical field, optical characterization of cells and tissues is a valuable tool for understanding physiological mechanisms. Current biomedical optical imaging techniques include fluorescence imaging [1], confocal microscopy [2], optical coherence tomography [3], two-photon microscopy [4], near-infrared spectroscopy [5], and diffuse optical tomography [6]. These techniques have significantly advanced biomedical technology and are widely used for both preclinical and clinical purposes. However, the strong optical scattering within turbid biological tissues fundamentally limits the imaging depth of these pure optical imaging techniques to no deeper than the optical ballistic depth (< 1 mm). Thus, their observation depth is superficial and other imaging modalities are needed to explore deeper layers of biological tissue [7].

Photoacoustic imaging (PAI), a promising biomedical technique, achieves superior imaging depths by forming images from optically-derived acoustic signals, which inherently attenuate less than optical signals in biological tissue [8–10]. PAI is based on the photoacoustic (PA) effect, in which energy is converted from light to acoustic waves via thermoelastic expansion [11–16]. To generate PA waves, a laser beam with a typical pulse width of a few nanoseconds illuminates the target tissue. The optical chromophores in biological tissue absorb the light energy and then release the energy soon after. The energy release can can occur as either light energy with a slightly shifted wavelength or as thermal energy that causes thermoelastic expansion. In PAI, the rapidly alternating thermoelastic expansion and contraction caused by pulsed light illumination generates vibrations in tissue that propagate as acoustic waves called PA waves. The generated PA waves can be detected by conventional ultrasound (US) transducers for image generation. Because PAI and ultrasound imaging (USI) share the same signal reception and image reconstruction principle, the two modalities are technically fully compatible and can be implemented in a single US imaging platform accompanied with pulse laser source [17–21]. Since PAI can capture the photochemical properties of the target site, combining PAI with USI can provide both chemical and structural information about a target tissue.

One distinctive advantage of PAI is that its resolution and imaging depth can be adjusted to suit a specific target area. The resolution of PA signals depends on both the optical focus of the excitation laser and the acoustic focus of the receiving US transducer [22], so images with tuned spatial resolutions and imaging depths can be achieved by modifying the system configuration [23]. PAI’s wide applications to date have included nanoscale surface and organelle imaging [24–28], microscale cellular imaging [29–32], macroscale small animal imaging [33–35], and clinical human imaging [36–38]. Preclinical PAI studies have investigated metabolic changes in the ear [39], eye [40–42], and brain [43–45], and have examined exogenous contrast agents [46–51], drug delivery monitoring [52–54], image-guided therapy [55–57], and phototherapy [58–60]. Human clinical PAI studies have focused on cancers of the thyroid [61–64], breast [65–69], prostate [70, 71], and skin [72–75], and on peripheral vascular system [76–78].

In a step beyond observing the appearance of biological tissue, systemic analysis of sequentially acquired PA images can identify additional useful functional biomarkers. For example, in optically characterizing chromophores, the pixel intensity of PAI is basically proportional to the local concentration, so PAI readily visualizes the morphology of a lesion rich with a target chromophore. Technical advances such as faster laser pulse rates, wavelength-tunable laser sources, and faster scanning schemes have enabled the acquisition of sequential PA images at high frame rates. With this capability, PAI has the potential to sensitively capture undiscovered metabolic changes in a living body. By analyzing the sequentially acquired PAI images, secondary functional biomarkers such as local tissue composition, blood flow, and metabolic or neural activity can be quantified and spatially mapped as functional images. Ultimately, depending on the photophysical or photochemical changes of the target chromophore, we will be able to better comprehend the molecular or functional information of biological tissues in vivo [79–81].

In this review, we briefly discuss the features of multiscale and functional photoacoustic imaging (fPAI). We then provide a comprehensive overview of the latest developments in fPAI at different scales, ranging from nanoscale to microscale and macroscale. Next, we classify fPAI studies according to these scales, so readers can readily identify appropriate potential applications for each scale. This approach will encourage readers to explore PAI research further, promoting additional advances.

## Multiscalability and functionality of photoacoustic imaging

### Multiscale photoacoustic imaging

**Fig. 1:**
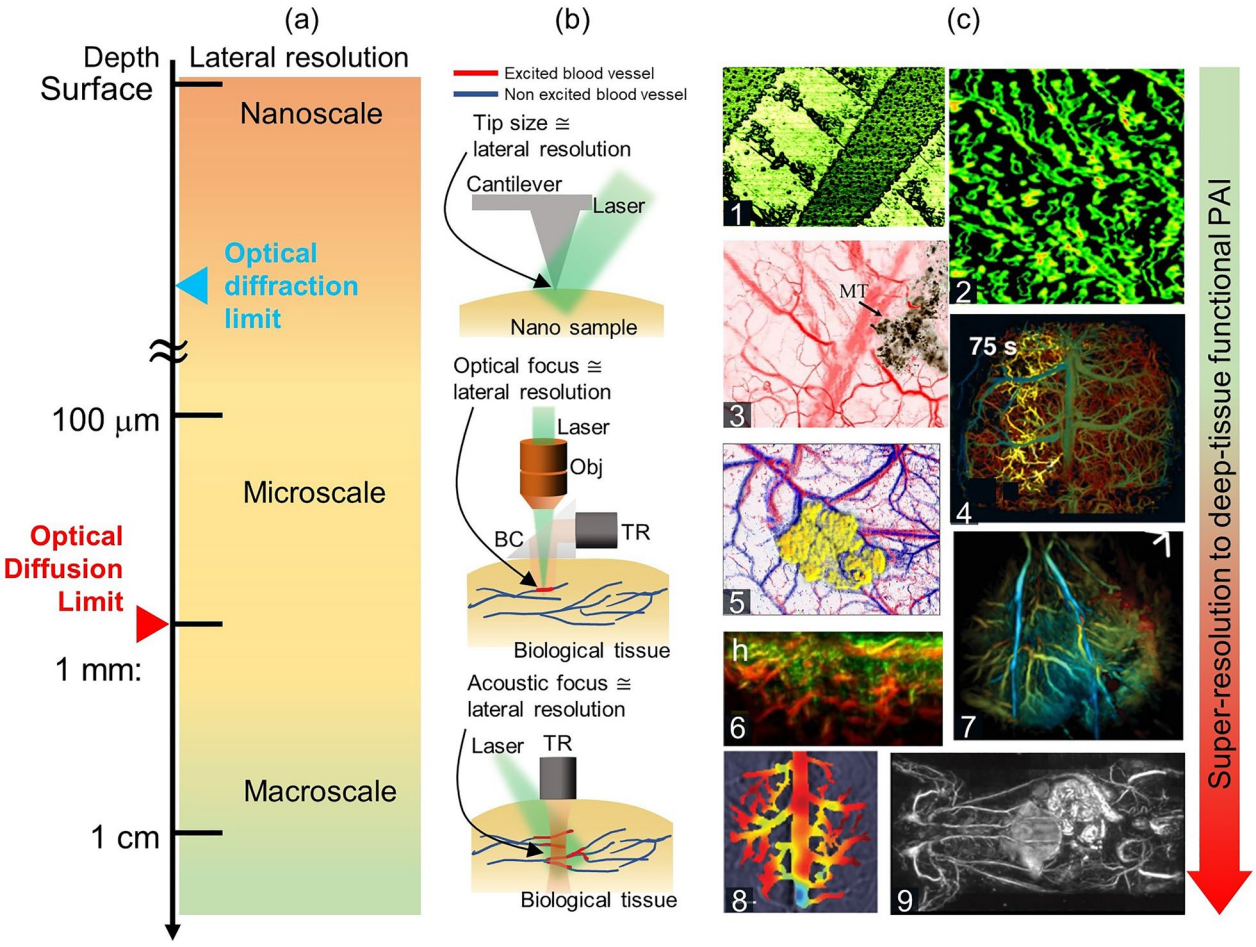
Characteristics of PAI, from surface to cm depth. **a** PAI depths, from the surface to one cm deep, with corresponding lateral resolutions. **b** Configuration of PAI for different scales. At nanoscale, microscale, and macroscale, the lateral resolutions are approximated by the sizes of the tip, optical focus, and acoustic focus, respectively. **c** Representitve super-resolution to deep-tissue functional PA images. Panels 1–9 respectively show an organic semiconductor crack, mitochondria, microvasculature with melanoma, brain vasculature and SO_2_ change, a blood vessel SO_2_ map with melanoma, human melanoma vasculature, the hemodynamics of a fatty liver, the hemodynamics of cerebral, and whole body vasculature, PAI, photoacoustic imaging; PA, photoacoustic; SO_2_, oxygen saturation; TR, ultrasound transducer; BC, beam combiner; Obj, objective lens. The images are reproduced with permission from [17, 24, 25, 82–87]

PAI uses optical excitation and acoustic detection, so its resolution can be either optically or acoustically determined, depending on the desired penetration depth (Fig. 1a). There are two main implementations of PAI [7, 88]: optical-resolution PAI and acoustic-resolution PAI. Optical-resolution PAI (i.e., optical-resolution photoacoustic microscopy, OR-PAM) can achieve high lateral resolution through fine optical focusing within the optical diffusion limit. The lateral resolution of OR-PAM is limited by optical diffraction, and can reach cellular and sub-cellular levels. Originally, OR-PAM had a lateral resolution of around 5 µm and an imaging depth of around 1 mm [89–92]. The lateral resolution has since been improved to around 220 nm (i.e., sub-wavelength photoacoustic microscopy, SW-PAM), allowing single red blood cells to be imaged [82, 93]. Nonlinear label-free PA nanoscopy has pushed the limits of OR-PAM further, achieving a lateral resolution of around 88 nm and a sub-µm axial resolution [25]. Furthermore, labeling the samples could potentially lead to further improvements in lateral resolution. With these improvements, OR-PAM has become widely applied in fields such as neurology [94, 95], vascular biology [96], dermatology [97, 98], and ophthalmology [99, 100].

Despite the fact that a series of OR-PAM instruments have garnered attention for their ability to achieve lateral resolutions beyond the optical diffraction limit, their restricted imaging depth has highlighted the constraints of optically-based imaging. Consequently, acoustic-resolution PAI is used for deep tissue imaging, taking advantage of its acoustically determined spatial resolutions [7]. It can be performed through raster scanning with a weakly focused optical illumination and a spherically focused US transducer (i.e., acoustic-resolution photoacoustic microscopy, AR-PAM) or by using wide-field optical illumination and parallel acoustic detection with an US transducer array (i.e., photoacoustic computed tomography, PACT). The spatial resolution and imaging depth of acoustic-resolution PAI are highly scalable with the US frequency, with lower frequencies achieving deeper penetration but lower resolution [7]. Typical lateral resolution and image depth values for AR-PAM are 20–50 µm and ~ 5 mm. The lateral resolution of PACT using an array-type transducer is 100–400 µm, and the image depth is ~ 8 cm. Photoacoustic macroscopy (PAMac) is a system that can bridge the gap between AR-PAM and PACT. As with AR-PAM, a single-element transducer is used for PAMac. Acoustic-resolution PAI has been successful in deep tissue imaging, such as small animal whole-body imaging [38], human breast cancer detection [101], melanoma stage analysis [72], and thyroid cancer screening [62, 64].

### Functional photoacoustic imaging

**Fig. 2:**
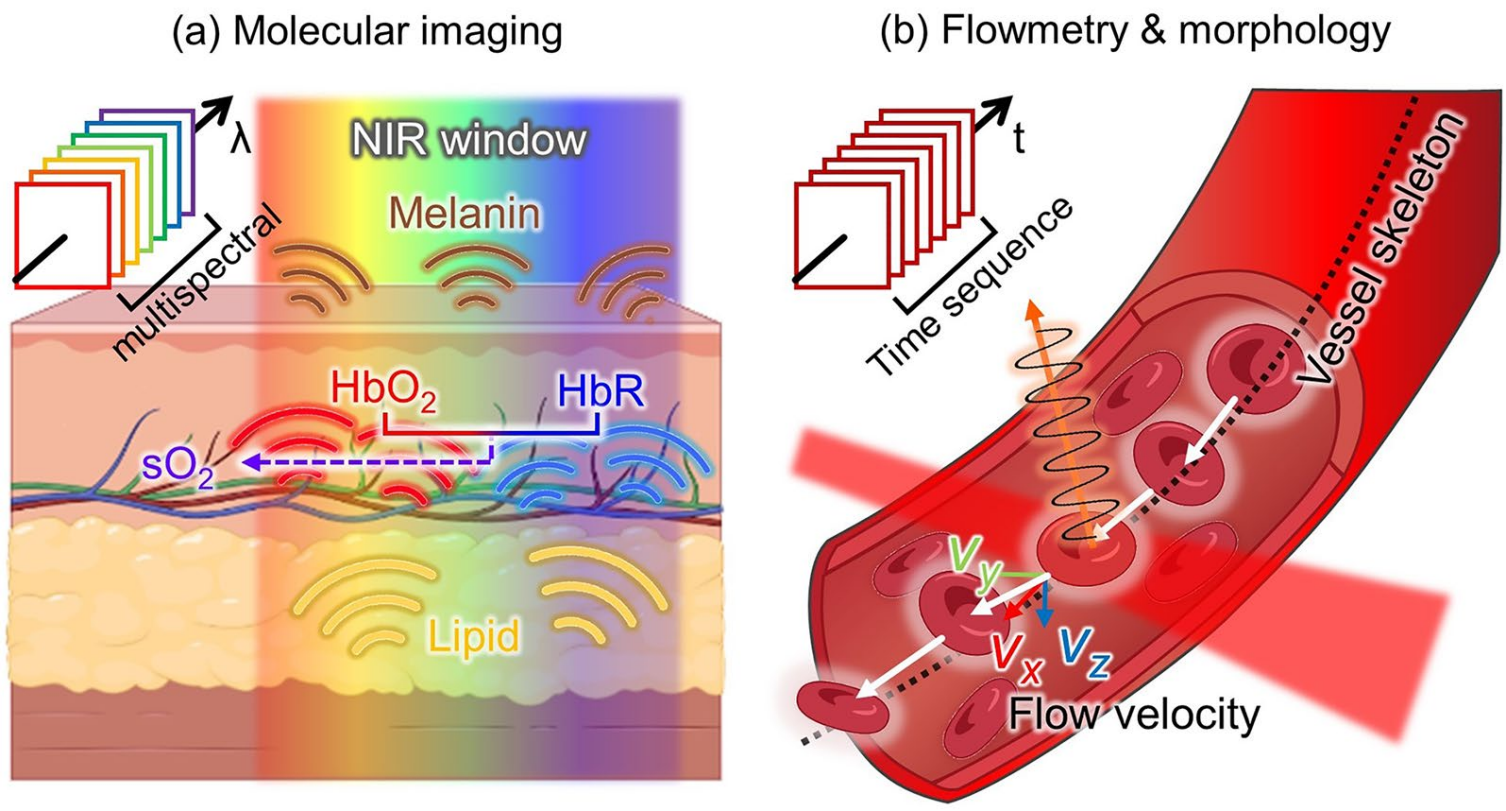
Principle of fPAI. **a** Molecular imaging using multiple light wavelengths to assess the characteristic photophysical PA spectra of biological chromophores. **b** Flowmetry and vessel morphology analyzed from time sequence image frames acquired at a high image frame rate. fPAI, functional photoacoustic imaging; λ, wavelength; NIR, near-infrared; HbO_2_, oxygenated hemoglobin; HbR, reduced hemoglobin; SO_2_, oxygen saturation; t, time; (v_x_, v_y_, v_z_), velocity constituents for the x, y, and z axes, respectively

The fPAI technique can measure various functional parameters in biological tissues, including the concentration of hemoglobin (HbT) [102, 103], SO_2_ [17, 104, 105] and blood flow [106, 107], in addition to vessel morphology and the metabolic rate of oxygen (MRO_2_) [108, 109]. These parameters can be roughly subdivided into two categories: local molecular composition parameters, and blood flowmetry or vessel morphology. Since each chromophore in biological tissue has a distinctive PA absorption spectrum within the NIR region, a distribution map of any given chromophore can be calculated by processing a series of PA images acquired via multiwavelength excitation (Fig. 2a) [110–112]. For instance, oxygenated hemoglobin and reduced hemoglobin can be distinguished by spectral unmixing, and the composition ratio of the two can directly enable calculating the oxygen saturation level and be used to distinguish arteries from veins.

Blood vessels are frequent targets of PAI, which can capture their morphology and blood flow features (Fig. 2b). Structural parameters such as vessel density [76, 113, 114], vessel diameter [113, 115], and vessel tortuosity [116–118] contribute to understanding the metabolic change and angiogenetic progression of a diseased organ or of carcinogenesis [119]. Further, blood flow can also be measured by observing PA signal fluctuations in sequential images acquired at a high frame rate [120, 121].

Dysfunctions in oxygen metabolism have been linked to various life-threatening diseases, and PA methods are ideal for imaging oxygen-metabolism in vivo. In particular, fPAI has proven valuable in diagnosing and evaluating cancerous tumors. For instance, Kim et al. utilized functional PAI to calculate the HbT and SO_2_ levels in the thyroid nodules of 52 patients (23 malignant cases and 29 benign cases), achieving 83% sensitivity and a 93% specificity in differentiating benign and malignant nodules [62]. As Fig. 1, illustrated, functional PAI is now used to image breast cancers [66, 101, 122, 123], prostate cancer [124–126], and melanoma [72, 73], and also diseases affecting the lymphatic system [127–129], brain [130–132], blood vessels [36, 37], and musculoskeletal system [133–135].

## Advances in functional photoacoustic imaging at different scales

### Nanoscale photoacoustic imaging, from the surface to organelles

**Fig. 3:**
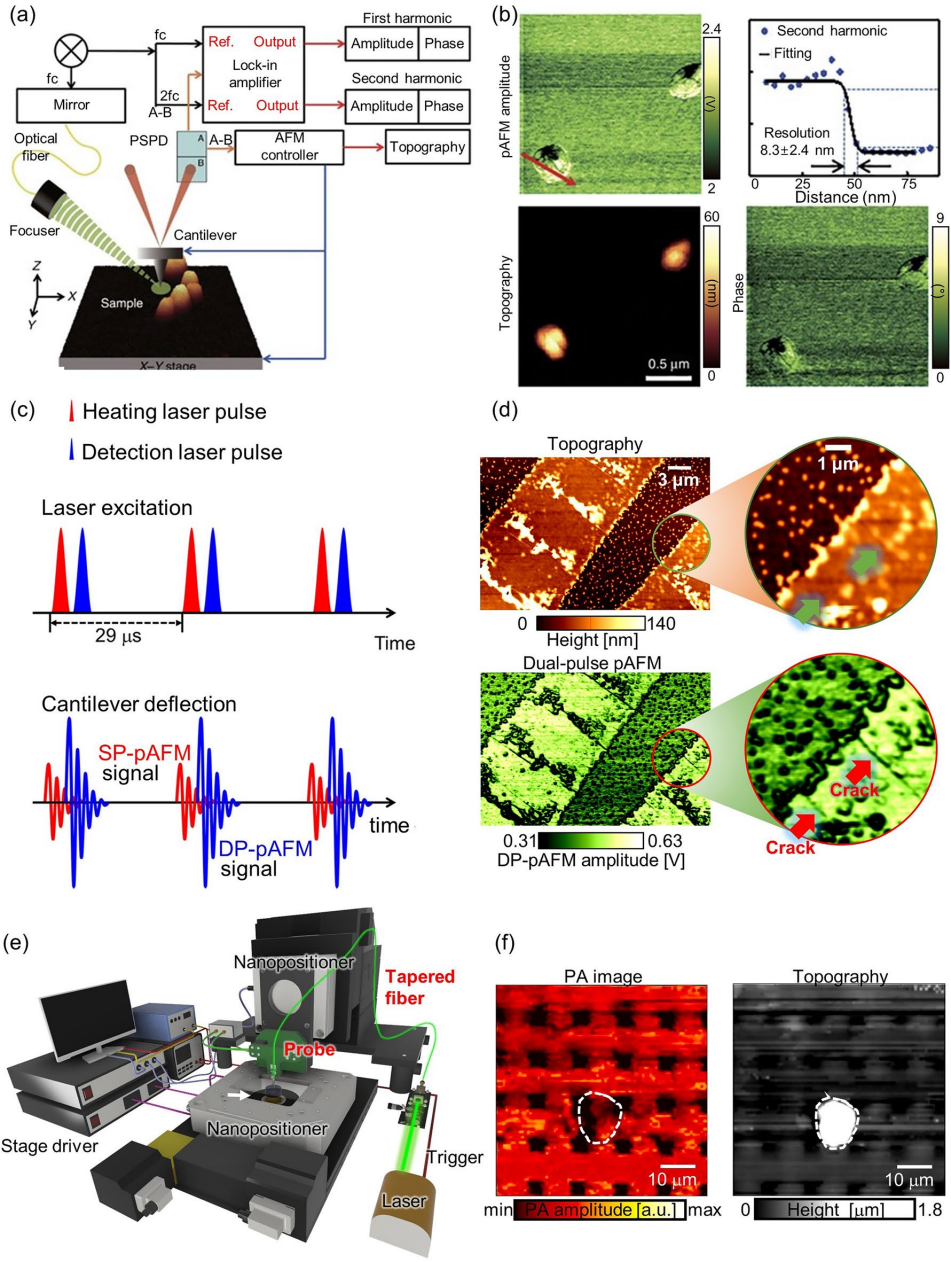
Super-resolution PAM techniques for surface imaging. **a** Schematic of a super-resolution visible pAFM. **b** A pAFM image, and topographic and phase images of single gold nanoparticles, with their lateral resolution. **c** Illustration of the dual-pulse mechanism for DP-pAFM signal generation. **d** Organic semiconductor topography and a DP-pAFM image for nanocrack analysis. **e** System configuration of NSPM. **f** Two-dimensional NSPM image and the topography of a gold microlattice. PAM, photoacoustic microscope; pAFM, photoactivated atomic force microscopy; SP-pAFM, single-pulse photoactivated atomic force microscopy. DP-pAFM, dual-pulse photoactivated atomic force microscopy; NSPM, near-field scanning photoacoustic microscopy; PA, photoacoustic. The images are reproduced with permission from [24, 27, 28]

Researchers are actively applying the strong light absorption contrast of PA to functional PA nanoscopy. As one example, the photoactivated atomic force microscopy (pAFM) presented by our group has demonstrated super-resolution capabilities, achieving a resolution of ~ 8 nm (Fig. 3a, b) [28]. In pAFM, which combines atomic force microscopy and a visible-range pulse laser, the sample is thermally expanded by the laser and induces vibration in the cantilever tip. The magnitude of this vibration is converted into an electrical signal called a pAFM signal. Super-resolution imaging of gold nanospheres, gold nanowires, and single cells has been achieved using this method. The functionality of pAFM was also demonstrated through the simultaneous acquisition of both height and phase information of samples (Fig. 3b). Our group has made an interesting contribution to the improvement of pAFM by applying a dual pulse mechanism [24]. This approach increases the SNR of pAFM by nonlinearly expanding the sample's thermal expansion by the irradiating it with two laser pulses separated by a nanosecond interval. This technique increases the vibration of the cantilever tip and a correspondingly increases the pAFM signal and SNR (Fig. 3c). In this study, nano cracks in organic semiconductors were clearly observed, which pose significant challenges for the semiconductor industry (Fig. 3d). Continued research on pAFM is contributing greatly to the development of super-resolution microscopes utilizing PA effects. A similar technology, atomic force microscopy-based infrared spectroscopy (AFM-IR) uses a visible wavelength laser to observe cells or organic semiconductors, AFM-IR uses an infrared laser to provide chemical analysis and compositional mapping with super resolution [136].

However, because pAFM or AFM-IR indirectly measures the PA effect in the sample through a cantilever tip, errors can be introduced by probe defects. Our group has presented a solution to the probe defect issue in a study on near-field scanning photoacoustic microscopy (NSPM) [27]. NSPM directly acquires PA signals by bringing a tapered optical fiber to within a few nm of the sample surface, without the use of a lens (Fig. 3e). Simulations confirmed the generation of evanescent waves at near fields, and experimental results showed the acquisition of near-field PA images of micro-scale gold lattice. NSPM provides the ability to simultaneously acquire not only light absorption information but also height information to analyze the morphological characteristics of a sample (Fig. 3f). This preliminary study represents a significant step towards the development of a super-resolution photoacoustic microscopy (SR-PAM).

**Fig. 4:**
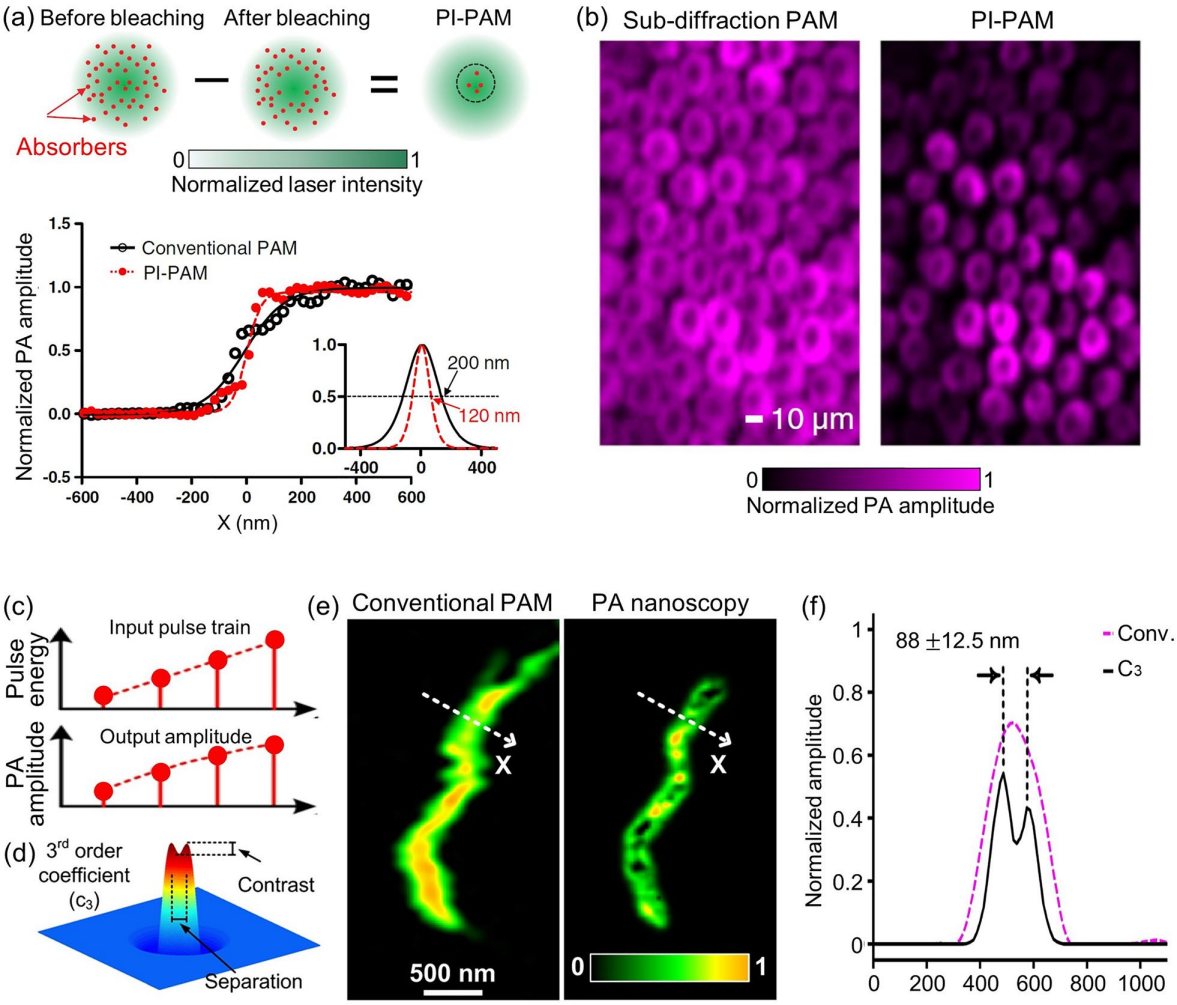
Far-field super-resolution PAM techniques for imaging single cells and organelles. **a** Principle of nonlinear photobleaching-based PI-PAM, depicting the lateral resolutions of PI-PAM and conventional OR-PAM along the edge spread function. **b** In vitro PI-PAM image of live rose petal epidermal cells. **c** Illustration of nonlinearity between pulse energy and resultant PA amplitude featured in nonlinear absorption-based PA nanoscopy. **d** Simulation of a 3rd order PA coefficient image which distinguishes two particles. **e** A mitochondrion imaged by conventional PAM and 3rd order PA nanoscopy. **f** Normalized PA amplitude along the dashed lines in panel **e**. The images are reproduced with permission [25, 26]

The imaging techniques discussed thus far, which enable near fieldsuper-resolution PAI, are useful only for a restricted area, such as the sample surface. Achieving far field super-resolution PAI can crucially broaden the range of samples that can be studied. Several efforts have been made to visualize biological tissues with a far-field super-resolution microscope using nonlinear mechanisms. Yao et al. proposed a new imaging technique, called photoimprint photoacoustic microscopy (PI-PAM), which uses a dual-pulse excitation to improve resolution [26]. In this technique, the first excitation pulse generates a PA signal throughout the entire excitation volume. However, due to the uneven illumination of the Gaussian beam, more absorbers in the central region are bleached than those in the peripheral region. During the second excitation pulse, molecules in the bleached central area have reduced absorption and generate a smaller PA signal than in the first excitation. The difference between the two signals is nonlinear to the excitation energy and represents the signal contribution from mainly the central focal area. This method improved both lateral and axial resolution (Fig. 4a). In particular, the lateral resolution was improved by a factor of $\sqrt{1+b}$, where $b$ is the photobleaching rate for excitation intensity. They demonstrated the effectiveness of PI-PAM by imaging live rose petal epidermal cells at 570 nm. Compared to conventional PAM, PI-PAM showed the cells more clearly (Fig. 4b). In other work, Danielli et al. reported PA nanoscopy utilizing nonlinear thermal expansion and optical absorption saturation [25]. Notably, the optical absorption coefficient saturates at higher optical fluence levels, providing nonlinear PA signals. High-order nonlinear PA images can be created by using an optical pulse train with different energies that hit the same target, improving the lateral resolution by a factor compared to conventional PAM (Fig. 4c). To demonstrate these high-order PA images, they simulated a Gaussian illumination of 226 nm and two 5 nm diameter targets located 90 nm apart. The third-order PA coefficient clearly distinguished the two targets (Fig. 4d). In addition, the PA nanoscopy technique was then applied to image a single mitochondrion, demonstrating superior image quality, with 88-nm lateral resolution, compared to conventional PAM (Figs. 4e, f). All studies reviewed in this section are summarized in Table 1.

**Table 1:** Super-resolution photoacoustic microscopy system specifications

Ref.	System scale	Functionality and application	Transducer		Laser		Lateral resolution	Imaging depth
			f (MHz)	Element	PRF	Wavelength (nm)		
[28]	SR-PAM	Nanoscale imaging, phase, topography	–	–	2 k	532	8 nm	Surface
[24]	SR-PAM	Nano crack identification	–	–	2 k	532	8 nm	Surface
[27]	OR-PAM	Microscale imaging, topography	35	1	1 k	532	1 μm	Surface
[25]	SR-PAM	Single mitochondria, cells	40 MHz	1	2.35 k	532	88 nm	Cellular
[26]	SR-PAM	Single cells	40 MHz	1	–	532	120 nm	Cellular

f, center frequency; PRF, pulse repetition frequency; OR-PAM, optical-resolution photoacoustic microscope; SR-PAM, super-resolution photoacoustic microscope; k, kHz

### Microscale photoacoustic imaging from a single cell to in vivo

**Fig. 5:**
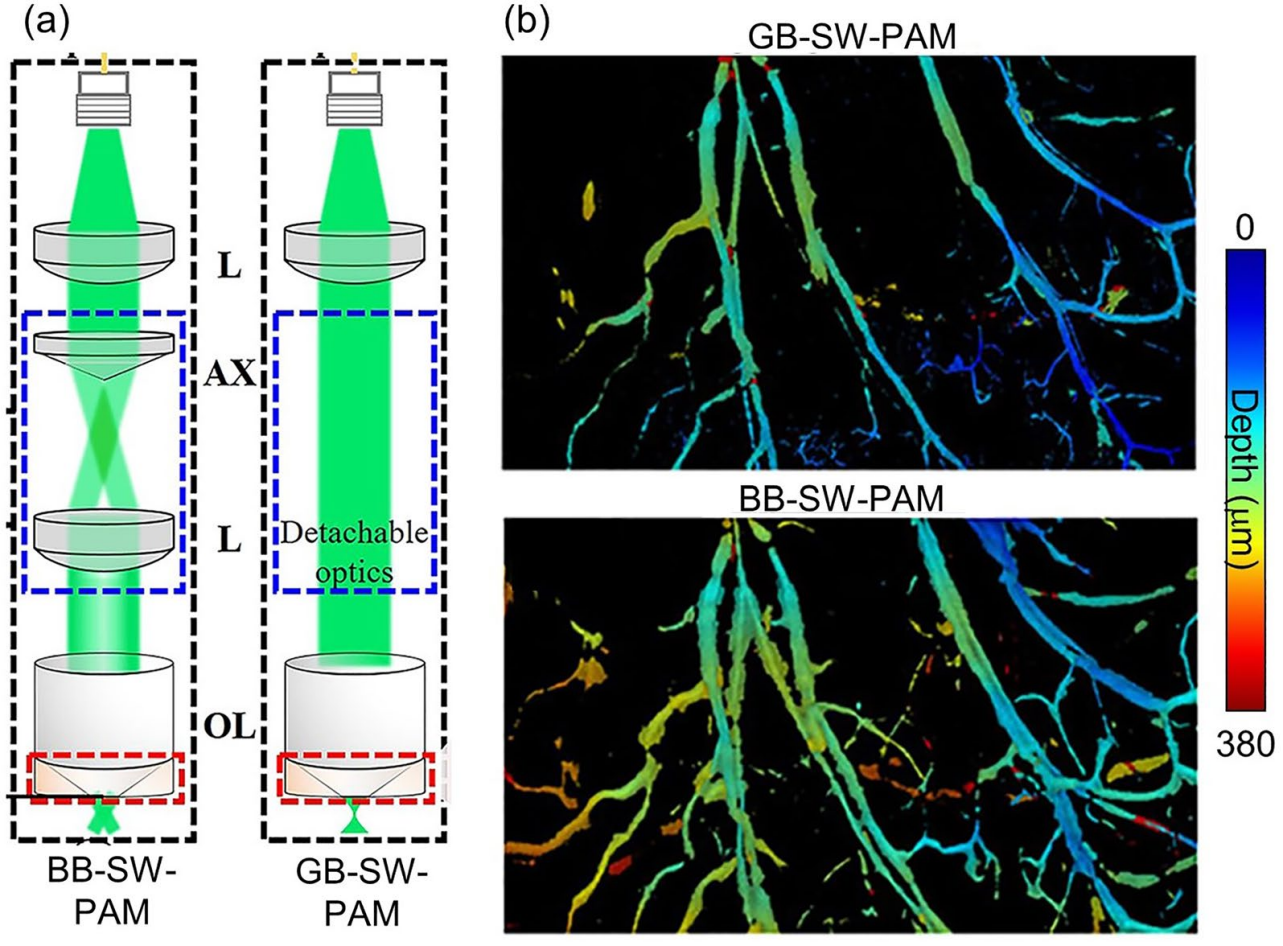
SW-PAM techniques for single cells and in vivo imaging. **a** Schematic of switchable BB-SW-PAM and GB-SW-PAM. **b** Comparison of PA microvasculature images obtained with GB-SW-PAM and BB-SW-PAM. BB-SW-PAM, Bessel-beeam sub-wavelength photoacoustic microscopy; GB-SW-PAM, Gaussian-beam sub-wavelength photoacoustic microscopy; OL, objective lens; L, lens; AX, axicon lens. The images are reproduced with permission from [93]

PA microscopy, whose resolution is determined by the optical focus within the optical diffusion limit (i.e., < 1 mm), is effective not only in obtaining high-resolution images but also in providing precise quantitative results. In particular, SW-PAM, with a lateral resolution close to the optical diffraction limit, can image single cells. This level of lateral resolution is typically achieved by using an objective lens with a numerical aperture (NA) of 1.0 or greater. The first SW-PAM system, presented by Zhang et al., used an objective lens with an NA of 1.23 and a 532 nm laser source to achieve a lateral resolution of 220 nm [82]. The system works in transmission mode, where the PA excitation and ultrasonic detectors are positioned in opposition across the sample. This SW-PAM system captured high-resolution images of single melanoma cells and red blood cells. Notably, it demonstrated the use of dual intrinsic contrasts by imaging blood vessels and a transplanted melanoma together in the ear of a mouse. Zhang's SW-PAM system has promising in vivo imaging capabilities and excellent lateral resolution, but its use of transmission mode limits the thickness of the samples that can be imaged. To overcome this limitation, Song et al. presented an SW-PAM system that employs reflection-mode imaging and maintains a sub-wavelength lateral resolution [137]. The system utilized an objective lens with an NA of 1.0 and a miniature ultrasonic transducer, which were combined in a custom-made transducer holder. To validate the reflection-mode SW-PAM system, the authors demonstrated high-resolution PA imaging of the vasculature in a mouse ear and further confirmed the ability to capture images of relatively deeper areas than possible with transmission-mode SW-PAM. They successfully demonstrated the implementation of reflection mode, which is challenging to achieve with a high NA objective lens. However, the inherently short depth-of-field associated with high NA objective lenses can limit the amount of information that can be obtained by an SW-PAM system. To address this issue, Park et al. used a Bessel beam with an axicon lens to generate an extended depth-of-field in reflection mode while maintaining sub-wavelength resolution [93]. Their system could be easily switched between Bessel-beam SW-PAM (BB-SW-PAM) and Gaussian-beam SW-PAM (GB-SW-PAM) to accurately measure the superior performance of BB-SW-PAM (Fig. 5a): the BB-SW-PAM exhibited a resolution of 300 nm while demonstrating a seven-fold improvement in depth of field compared to GB-SW-PAM (Fig. 5b). Using in vivo mouse ear vasculature imaging, they demonstrated that BB-SW-PAM can obtain vascular information at a greater variety of depths than GB-SW-PAM.

**Fig. 6:**
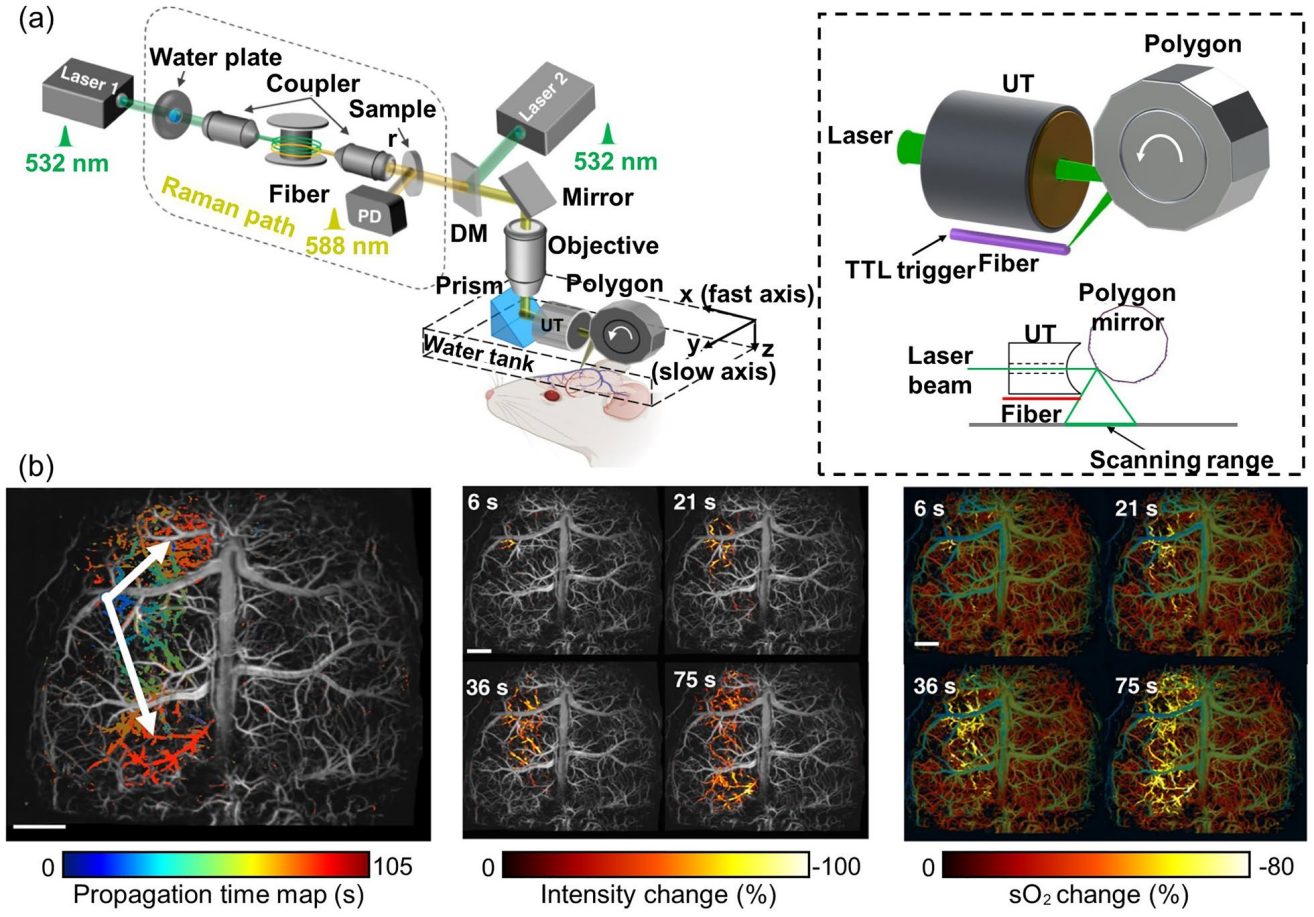
Ultrafast wide-field, high-resolution, functional OR-PAM for the study of ischemic stroke. **a** Schematic of the ultrafast functional PAM. The 532 nm light source and 558 nm Raman source are combined for functional imaging. Inset illustrates the UT, polygon scanner, laser, and scanning range. **b** Propagation time map of the SD wave and changes in PA intensity and SO_2_ during the SD wave propagation at different time steps. PAM, photoacoustic microscopy; DM, dichroic mirror; UT, ultrasound; SO_2_, oxygen saturation; PA, photoacoustic; SD, spreading depolarization. The images are reproduced with permission from [83]

Although SW-PAM’s achievement of a resolution close to the optical diffraction limit has shown promising results in cellular imaging, the system’s short focal length, structural limitations, and slow imaging speed make it challenging to achieve a more diverse functional PA imaging. However, OR-PAM has a longer focal length, allowing for a relatively more flexible system configuration and a significantly faster imaging speed, albeit at a lower resolution than SW-PAM. Recently, thanks to its fast imaging speed and functional capabilities, OR-PAM has been used for precise quantification based on high-resolution imaging. For example, by combining a contour scanning technique and a functional OR-PAM with a 2 μm lateral resolution, Yeh et al. confirmed that multiple parameters of a single micro vessel can be quantified with a high signal-to-noise ratio (SNR) [138]. Further, functional OR-PAMs are important in understanding the physiological properties of diseased small animals [45]. As an example, high-resolution functional PAI has been used to evaluate small animals manifesting symptoms of stroke [139]. Stroke is the leading cause of death and disability in patients with ischemic heart disease. Using high-resolution PAI to characterize the cerebrovascular properties in stroke-induced small animals may help to understand stroke in humans. Recently, to study ischemic stroke, Zhu et al. developed ultrafast wide-field OR-PAM (UFF-PAM), capable of real-time high-resolution whole-brain imaging of hemodynamics and oxygenation [83]. The authors emphasized the engineering innovations of UFF-PAM, such as SRS-based dual-wavelength laser excitation, a 12-facet-polygon scanner, and deep-learning-based image upsampling (Fig. 6a). The volumetric imaging rate, lateral resolution, and field-of-view (FOV) of UFF-PAM were 2 Hz, 10 µm, and 11 mm, respectively. To observe the functional response of microvasculature to stroke-induced spreading depolarization (SD) waves, they prepared a mouse model in which permanent stroke was induced in the left hemisphere by completely occluding the left carotid artery, and then temporarily occluded the right carotid artery. With the large FOV, high speed, and high resolution of UFF-PAM, the researchers were able to calculate the average SD wave speed, to localize the origination point of each wave, to map the spreading direction, pattern, duration, and affected area (Fig. 6b), and to quantify vasoconstriction and the decrease in local oxygen saturation. Cao et al. used a functional OR-PAM to conduct an interesting study of the neuroprotective effect of sphingosine 1-phosphate (S1P), a bioactive metabolite of sphingolipids, against ischemic stroke in awake mice [140]. The authors increased the blood S1P levels by administering sphingosine kinase 2 (SPK2) to the mice and evaluated the resulting changes in cerebral hemodynamics and oxygen metabolism. The results showed a restoration of SO_2_ levels in hypoxic conditions after SPK2 injection, but no significant changes in blood flow or HbT. These findings suggest that elevated blood S1P levels may lead to neuroprotective effects against ischemic stroke.

Diabetes is highly correlative with cardiovascular disease such as high blood pressure or stroke since sustained high blood glucose level constantly damages the blood vessels.

Krumholz et al. used functional OR-PAM to photoacoustically image diabetes-induced damage to the microvasculature and confirmed the possibility of diabetes diagnosis [141].

**Fig. 7:**
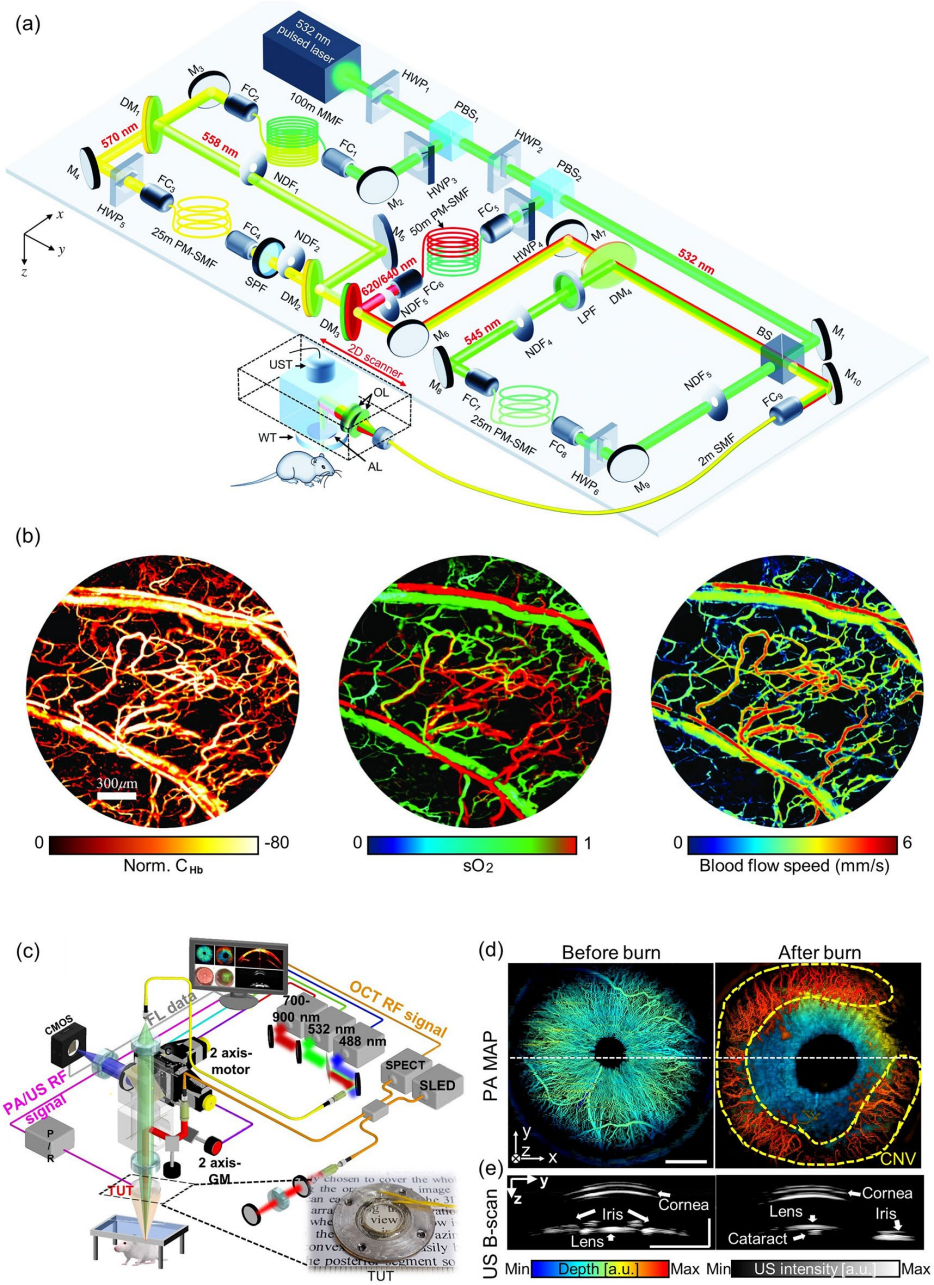
High-resolution and functional OR-PAM for the study of cancer. **a** Schematic of a functional OR-PAM using a five-wavelength pulsed laser and stimulated Raman scattering, **b** hemoglobin concentration, oxygen saturation, and blood flow speed imaging results from the ear of a tumor-implanted mouse. **c**–**e** Quadruple imaging system based on a TUT. **c** System configuration. **d** PA depth encoded MAP images and **e** cross-sectional US B-scan images before and after the chemical burns. OR-PAM, optical-resolution photoacoustic microscopy; C_Hb_, total hemoglobin; SO_2_, oxygen saturation; HWP, half-wave plate; LPF, long-pass filter; PBS, polarizing beam splitter; M, mirror; FC, fiber coupler; PM-SMF, polarization-maintaining single-mode fiber; SMF, simgle-mode fiber; SPF, short-pass filter; DM, dichroic mirror; NDF, neutral density filter; OL, optical lens; AL, acoustic lens; WT, water tank; UST, ultrasound transducer; BS, beam splitter. PA, photoacoustic; US, ultrasound; TUT, transparent ultrasound transducer; CMOS, complementary metal oxide semiconductor; SLED, superluminescent light-emitting diode; SPECT, spectrometer. The images are reproduced with permission from [17, 142]

Cancer is a major global health issue and a significant cause of death. To develop new cancer diagnostic tools and therapies and improve patient outcomes, it is crucial to study the growth and treatment of tumors in animal models. Specifically, the angiogenesis around cancers and the hypoxia/hyperoxia in peripheral blood vessels are important factors in determining the occurrence and malignancy of cancer. Liu et al. developed a 5-wavelength pulse laser system based on stimulated Raman scattering to provide functional information about blood vessels, such as HbT, SO_2_, and blood flow speed, and applied it OR-PAM [142]. Wavelengths of 532, 545, 558, 620, and 640 nm were utilized to image blood and lymphatic vessels, and the blood flow speed was measured using the dual-pulse technique (Fig. 7a). The SO_2_ was calculated using the Gruneisen-relaxation effect. The researchers observed angiogenesis, a hallmark of cancer, in the ear of a mouse with a transplanted tumor and quantified the consequent increases in SO_2_ and blood flow speed, which accurately reflected the rapid growth of an early-stage tumor (Fig. 7b). Gong et al. used functional OR-PAM to investigate the impact of glioma on the vascular structure and function of the mouse brain [143]. They found that glioma causes microvessel growth that increases the vascular proportion in the cerebral cortex. They also confimed that the glioma causes loss of response in one hemisphere and abnormal response in the other, degrading functional connectivity.

OR-PAM has also shown great promise in studying ophthalmic diseases [42, 99, 144–146]. Recently, a fascinating research endeavor introduced a quadruple imaging system that combines OR-PAM, ultrasound imaging (USI), optical coherence tomography (OCT), and fluorescence imaging (FLI) [17]. This innovative system employed a transparent ultrasound sensor (TUT) and was specifically applied to corneal neovascularization (CNV) and inflammation casued by chemical burns (Fig. 7c). The study acquired quadruple images to analyze morphological and physiological responses. Results showed that PA images pre- and post-chemical burn revealed the presence of CNV by scrutinizing depth-encoded maximum amplitude projection images (Fig. 7d). OCT successfully rendered the cornea visible by elucidating its structural layers. Furthermore, USI demonstrated its capability to induce morphological alterations in the eye, including the development of cataracts within the lens, potentially contributing to vision impairment (Fig. 7e). In the context of FLI, the visualization of corneal epithelial inflammation, a potential cause of corneal edema, was achieved through fluorescein staining. By offering a comprehensive range of multimodal functional images, this proposed system exhibits significant potential for application in diverse biomedical studies, promising substantial contributions to the field. All the studies reviewed in this section are summarized in Table 2.

**Table 2:** Summary of the microscale photoacoustic imaging system specifications

Ref.	System scale	Functionality and application	Transducer		Laser		Lateral resolution	Imaging depth
			f (MHz)	Element	PRF	Wavelength (nm)		
[82]	SW-PAM	Single cells, blood vessels and melanoma	40	1	–	532	220 nm	a hundred of nm
[137]	SW-PAM	Microvasculature network	42.6	1	40 k	532	320 nm	a hundred of nm
[93]	SW-PAM	Microvasculature network	41	1	–	532	270 nm	hundreds of nm
[83]	OR-PAM	Whole brain, oxygen saturation, stroke	40	1	800 k	532, 558	7 μm	1.5 mm
[140]	OR-PAM	Oxygen saturation, stroke	35	1	–	532, 558	–	–
[141]	OR-PAM	Diabetes, oxygen saturation, blood Fow	50	1	–	532, 561	3.4 μm	–
[142]	OR-PAM	Oxygen saturation, lymph mapping, blood flow speed, cancer	50	1	4 k	532, 545, 558, 570, 620/640	3.1 μm	0.8 mm
[143]	OR-PAM	Fractional change, glioma	–	1	600 k	532	11.8 μm	–
[17]	OR-PAM	Quadruple images, ophthalmic diseases	31.5	1	–	532	2.4 μm	–

f, center frequency; PRF, pulse repetition frequency; SW-PAM, subwavelength photoacoustic microscope; OR-PAM, optical-resolution photoacoustic microscope; k, kHz

### Macroscale photoacoustic imaging in deep-tissue applications in vivo

Macroscale PAI, which includes PA macroscopy (PAMac) and PACT, can expand the technical advantages of functional microscale PAI beyond shallow depths and a limited field-of-view to encompass a wider range of depths and larger fields-of-view. However, this comes at the cost of decreased resolution due to the larger scale of observation. PA macroscopy, the simplest configuration of macroscopic PAI, uses a motorized scanner to perform 2D raster scanning of a mechanically focused single-element transducer. Combined with a relatively simple image reconstruction algorithm, this type of system can provide volumetric microvasculature images of small animals or humans.

**Fig. 8:**
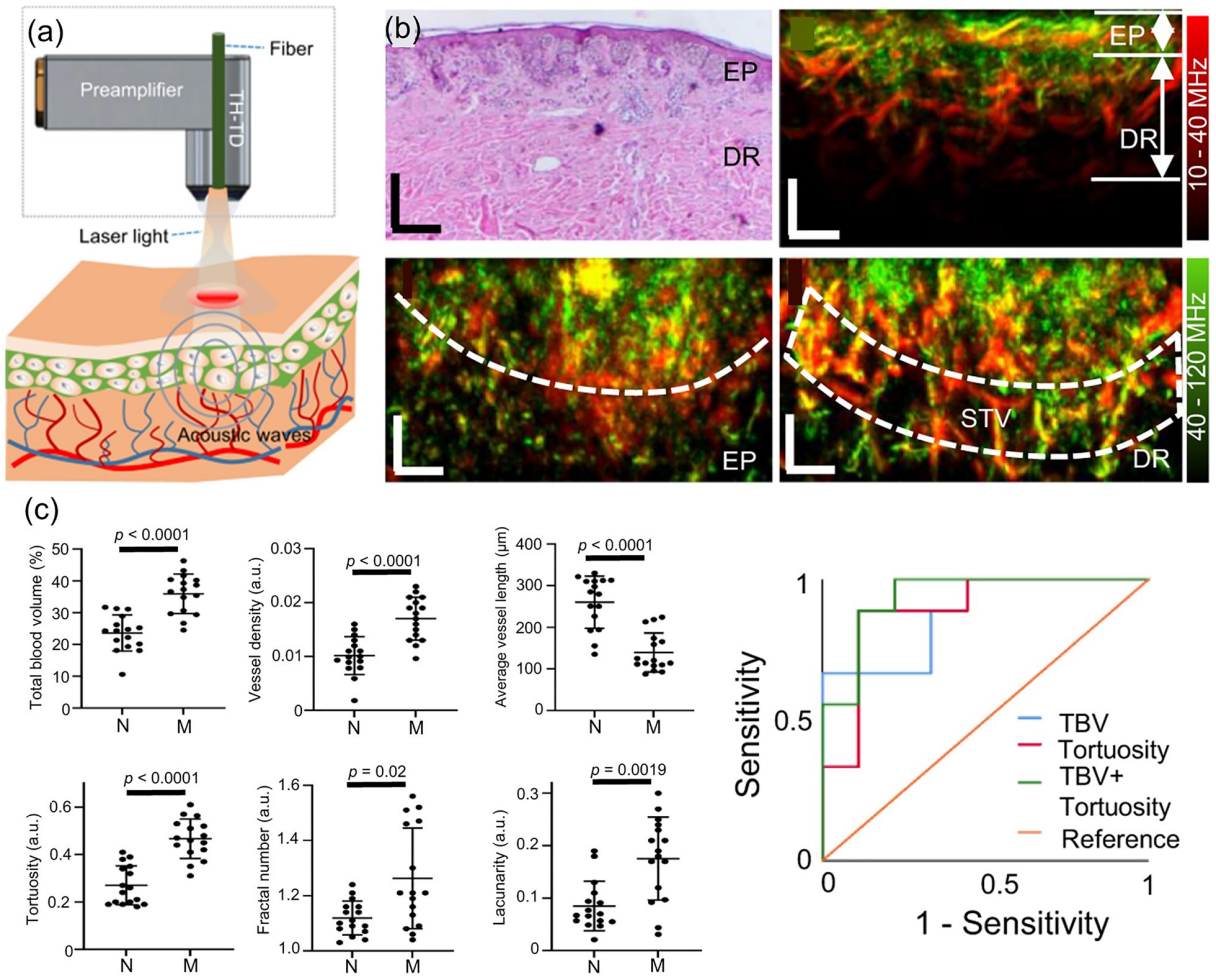
Single-breath-hold scan of human melanoma vasculature in vivo with accelerated FRSOM. **a** Schematic of a through-hole transducer implementation in an FRSOM setup. **b** Histological image (top, left) and cross-sectional MIP images (top, right) of human melanoma in vivo. The coronal-direction MIP at epidermal depth range (bottom, left) and dermal depth range (bottom, right) visualize the separation boundary between pigmented lesion and surrounding skin tissue, and surrounding tissue vessels beneath extending toward healthy skin tissue, respectively (marked with white dashed line). **c** Statistical comparison of computed vessel biomarkers between a normal nevus and melanoma lesions, and resultant ROC plots of melanoma differentiation with various biomarker combinations. All scale bars = 500 μm. TH-TD, through-hole transducer; EP, epidermis layer; DR, dermis layer; STV, surrounding tissue vessels; N, nevi; M, melanoma; a.u., arbitrary unit; TBV, total blood volume. The images are reproduced with permission from [85]

The center frequency of the transducer must be carefully selected to suit the feature being imaged. A high frequency transducer can provide the fine spatial resolution needed to image cutaneous microvascular structures. Raster-scan optoacoustic mesoscopy (RSOM) can produce microvasculature images from as deep as the subcutaneous bed, with tens of microns resolution. Using wavelength-tunable high repetition rate laser, PA images of a layer of tissue acquired at series of distinct wavelengths can be spectrally unmixed according to its chromophores, such as melanin, oxy-hemoglobin, and deoxy-hemoglobin, and the composition ratio between two hemoglobin distribution may spatially encode oxygen saturation map. In a clinical application, Li et al. used multispectral RSOM to track morphological and oxygenation changes in severe atopic dermatitis [147]. The clinical applicability of RSOM was demonstrated by comparative analysis of changes in the SO_2_ and epidermal thickness of skin microvessels before and after treatment. RSOM’s scanning speed and SNR were further increased by using a through-hole broadband transducer, enabling four times higher pulse energy and utilizing a full 1.4 kHz laser repetition rate [85]. As a consequence, a 4 × 2 mm^2^ field of view could be swept within a single breath-hold (~ 15 s), reducing motion artifacts and increasing temporal sensitivity (Fig. 8a, b). Statistical classification of cuteneous melanomas (n = 10) and benign nevi (n = 10) in vivo was demonstrated by using vessel morphology biomarkers, such as the total blood volume, average vessel length, tortuosity, and fractal number (Fig. 8c).

To image organs located more deeply beneath the skin, a lower frequency transducer should be selected, because low frequency PA signals are less attenuated in propagating through tissue. Low frequency systems are widely applied in studies where PA contrast agents have accumulated in deep targeted organs [148, 149]. Jeon et al. successfully demonstrated a multiscale whole-body PAMac setup for in vivo small animal study by switching between 5 and 40 MHz ultrasound transducers depending on the size of target organ [150]. Further, whole body multispectral PA images with 5 MHz transducer setup characterized the spectral features of major blood vessels as well as intact internal organs in vivo [151–155]. As a representative result, Lee et al. demonstrated dual-color PA images acquired after contrast agents were injected to delineate two different optically transparent lymphatic networks, which are invisible in PA images [156]. Recently, Park et al. updated the system to acquire PA and US images simultaneously [157]. In a follow-up study, Lee et al. successfully compensated for breathing movement during the raster scanning by using the structural information in USI [158]. In particular, three-dimensional (3D) SO_2_ distributions in the whole body of mice were successfully visualized. All studies reviewed in this section are summarized in Table 3.

**Table 3:** Summary of functional PA macroscopy systems’ specifications

Ref.	System scale	Functionality and application	Transducer		Laser		Lateral resolution (μm)	Imaging depth (mm)
			f (MHz)	Element	PRF	Wavelength (nm)		
[147]	PAMac	oxygen saturation	50	1	1.3 k	532, 555, 579, 606	40	2
[85]	PAMac	Melanoma; total blood volume, vessel density, average vessel length, tortuosity, fractal number, lacunarity	10–40, 40–120	1	1.4 k	532, 555, 576, 606	16.4	1.5
[150]	PAMac	multispectral whole body	5, 40	1	10	532, 700, 850, 1064	590	30
[156]	PAMac	dual-color lymph mapping	5	1	10	707, 860	590	30
[158]	PAMac	oxygen saturation	5	1	10	750, 800, 850	590	30

f, center frequency; PRF, pulse repetition frequency; AR-PAM, acoustic-resolution photoacoustic microscope; PAMac, photoacoustic macroscope

Despite improvements in its speed, mechanical scanning inescapably takes a long time (typically ~ 30 min for the whole body of a mouse), and its fixed focal depth yields to non-uniform lateral resolution. PACT, a superior system, uses electrical scanning and beamforming (i.e., receive focusing) with a multi-element array transducer to enable macroscale PAI to perform real-time tomography at a higher spatial resolution [159–164]. To deliver high energy laser pulses and provide better flexibility at clinical environment, laser pulse is delivered via multiple large core optical fibers or an optical fiber bundle instead of single core or free-space delivery optics. Linear or convex array transducers, which are already used in clinical ultrasound systems, are adequate for clincally translatable bimodal PA and US imaging. Arc-shaped or spherical array transducers can reconstruct volumetric PA images since the probe geometry can overcome the limited view effect arising from each element’s directivity [165–170].

Derived from the distinctive spectral response of hemoglobin throughout the NIR band, hematoscopic features such as the local hemoglobin concentration or oxygen saturation map are among the most useful functional biomarkers within multispectral PAI. They are calculated by serial PA image acquisition with a series of distinct wavelengths, preferrably at a high frame rate to prevent interframe motion artifacts and yield better spectrally unmixed results. However, the number of piezoelectric detectors in volumetric PA probes can be several times greater than the number of DAQ channels, and hence most volumetric imaging systems adopt a N-to-1 multipliexed channel connection. Consequently, multiple laser excitations are required to acquire a single PA image, which lowers the PA image frame rate and ultimately degrades the quality of functional PAI by inducing spatial errors or artifacts.

**Fig. 9:**
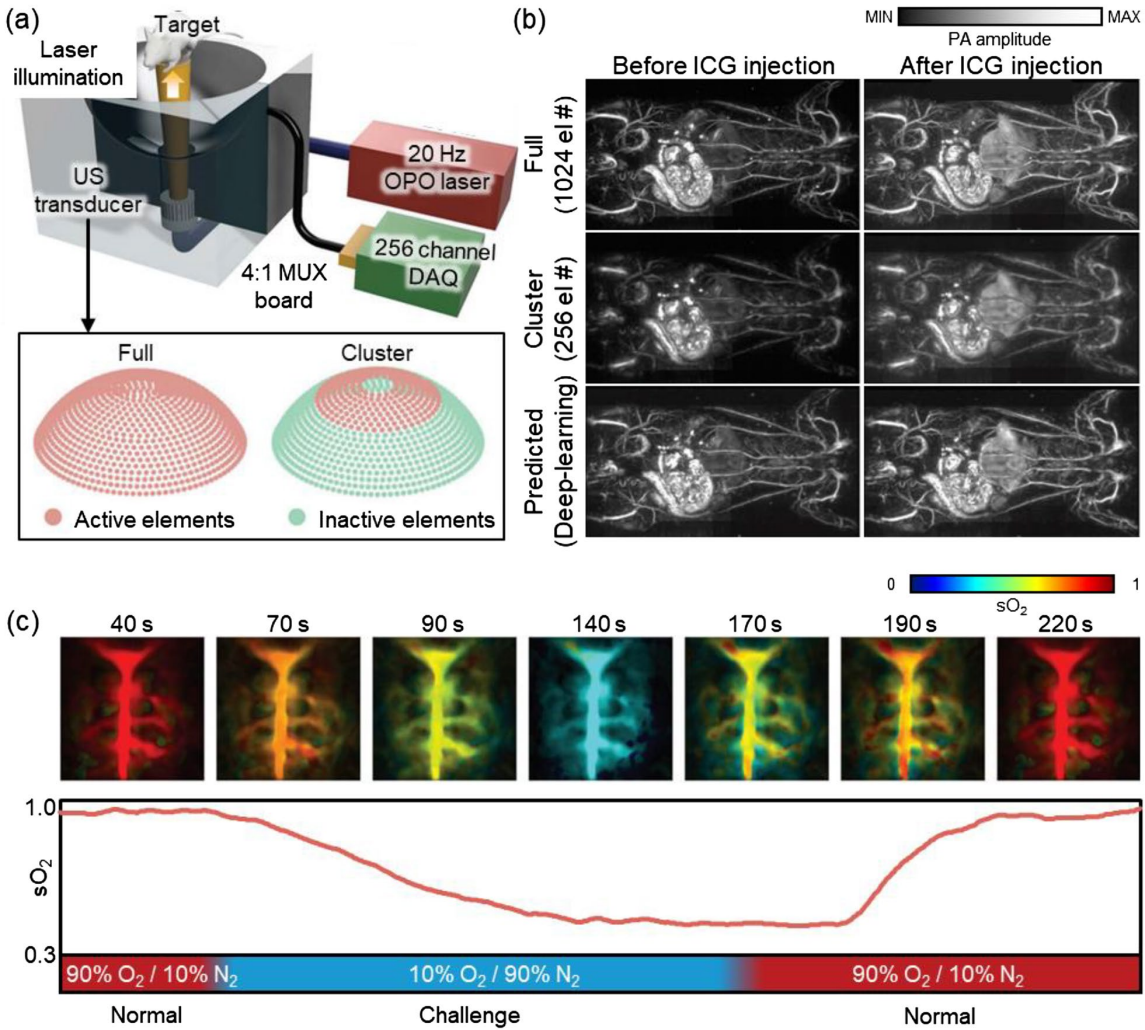
A deep-learning-assisted PAI system and its results. **a** Schematic illustration of a PAI system with a down-up oriented 1024-element spherical array transducer. Inset depicts active and inactive elements in the transducer in each acquisition mode. **b** Rat whole-body images in vivo before and after intravenous injection of ICG solution. Top row, with full data acquisition (4 laser shots, 1064 elements); middle row, with cluster data acquisition (1 laser shot, 256 elements); bottom row, predicted images generated by a deep-learning algorithm. **c** Spectrally unmixed SO_2_ distribution of the brain in rats breathing different mixtures of O_2_ and N_2_ gas in alternation. PAI, photoacoustic imaging; US, ultrasound; OPO, optical parametric oscillator; DAQ, data acquisition module; MUX, multiplexer; ICG, indocyanine green; SO_2_, hemoglobin oxygen saturation; O_2_, oxygen; N_2_, nitrogen. The images are reproduced with permission from [87]

Recently, Choi et al. demonstrated deep-learning-assisted image reconstruction to overcome temporal resolution loss coming from multiplexed channel connection [87]. Their system consisted of a tunable optical parametric oscillator (OPO) laser, a 1024-element spherical array transducer, and a 256-channel data acquisition module (Fig. 9a). The system originally required four laser illuminations to capture data from all the elements. To improve the temporal resolution of the system, they trained a deep-learning algorithm to improve the quality of the PA images obtained from the partial (256 elements) data, making it substantially comparable with the original images reconstructed with the full data (Fig. 9b). The enhanced temporal resolution was demonstrated by monitoring the brain hemodynamics in rats breathing different mixtures of oxygen and nitrogen in alternation (Fig. 9c). The spectrally unmixed SO_2_ map showed that oxygen levels decreased under the oxygen challenge (10% O_2_, 90% N_2_) and recovered shortly after the normal mixture (90% O_2_, 10% N_2_) was restored.

**Fig. 10:**
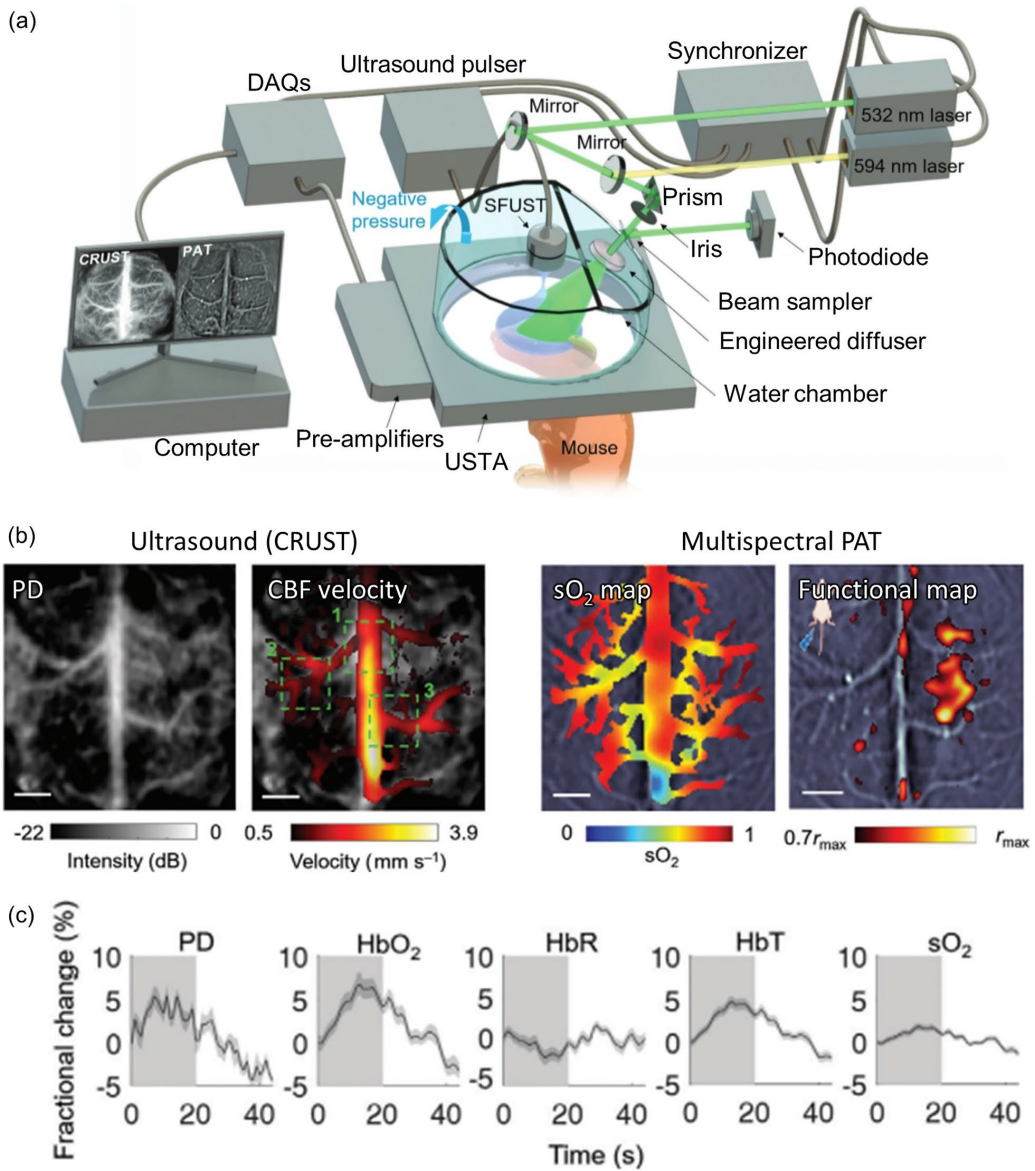
CRUST-PAT synergistically characterizes the cerebral hemodynamics of a mouse brain in an intact skull and scalp. **a** System configuration of CRUST-PAT. **b** Graphical mapping of biomarkers acquired by bimodal CRUST and multispectral PAT. **c** Computed fractional changes of PD, hemoglobin concentrations, and SO_2_ signals in response to hindlimb electrical stimulation. PD: power Doppler; CBF: cerebral blood flow; SO_2_:oxygen saturation; HbO_2_: oxygenated hemoglobin; HbR: reduced hemoglobin; HbT: total hemoglobin. The images are reproduced with permission from [86]

PAI, the only functional optical imaging modality that can provide blood oxygenation information at depths below the optical diffraction limit, is simultaneously operable with other medical imaging modalities. Because PAI is a hybrid acoustic imaging technique, it can provide complementary photochemical circulatory information when joined with simultaneous ultrasound imaging, a superior tool for capturing tomographic structure or flowmetry via ultrasound Doppler imaging. By utilizing the benefits of both modalities, Na et al. presented a combination of simultaneous cross-ray ultrasound tomography (CRUST) and PACT that used the same ring-shaped array to synergistically characterize cerebral hemodynamics in an intact skull and scalp [86]. To counteract the low transmission capability of the PA-purposed transducer and avoid cross-talk, a transmission-only single element spherically-focused transducer was placed in the central axis of the ring array. The passively received US RF signal was reconstructed into US images. A 4-kHz ultrafast pulse repetition rate was employed to detect the faint US Doppler signal from the cerebral microvascular flow (Fig. 10a). The US blood signal was spatiotemporally filtered out from the motionless tissue signal, then it was further processed into both power Doppler (PD) images and estimated blood vector flow map. Dual wavelength PAI acquisitions were interleaved in between of two US pulse train acquisition, under wavelength of 532 nm and 594 nm at 10 Hz pulse repetition, respectively, and further processed to obtain the estimated local concentrations of HbO_2_ and HbR (Fig. 10b). The local activation at ipsilateral and contralateral sites of the mouse brain during repeated hindlimb electrical stimulation was well demonstrated in both the US and PAI activation maps (Fig. 10c). Additionally, functional hemodynamic changes in PD, HbO_2_, HbR, HbT and SO_2_ were well synchronized to the external stimulation.

**Fig. 11:**
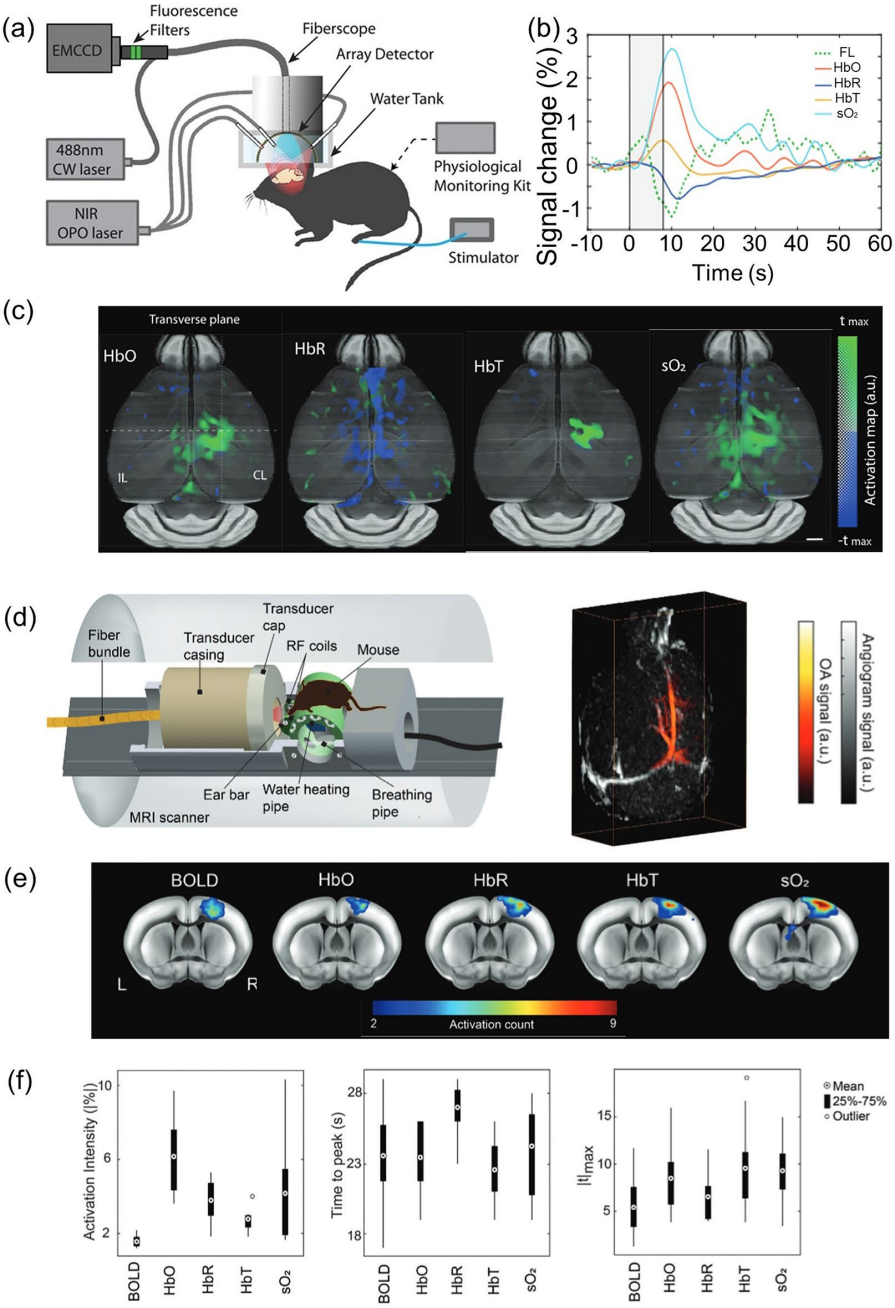
Simultaneous observation of neurovascular hemodynamic activity using fluorescence optoacoustic tomography (FLOT) and magnetic resonance optoacoustic tomography (MROT). **a** Configuration of the hybrid FLOT system for imaging mouse brain activation. **b** Average fractional changes of biomarkers—fluorescence, HbO_2_, HbR, HbT, and SO_2_—in response to an electrical paw stimulation burst. **c** Functional optoacoustic activation maps in transverse view corresponding to each biomarker. **d** The hybrid MROT system schematic with a representative 3D-coregistered MR angiogram and PA tomographic volume. **e** Activation count maps overlaid on the mouse brain atlas for MROT biomarker changes. **f** Statistical distributions of neural response parameters from the somatosensory forelimb region in the averaged time-courses. CW, continuous wave; NIR, near-infrared; OPO, optical parametric oscillator; FL, fluourescence; HbO, oxygenated hemoglobin; HbR, reduced hemoglobin; HbT, total hemoglobin; SO_2_, oxygen saturation; t_max_, time-to-maximum; OA, photoacoustic; BOLD, blood oxygen level-dependent signal. The images are reproduced with permission from [171, 172]

To understand the functional and molecular information underlying brain activity, sensing neural activation with GCaMP-mediated fluorescence imaging (FLI) has been widely employed. However, the shallow imaging depth impedes a full understading of brain physiology and neccesitates invasive interventions, such as craniectomy. By using functional PACT as an alternative tool to visualize brain hemodynamics in the intact skull, we can correlate PACT-derived and GCaMP FLI-derived biomarkers to potentially extend our knowledge of neurovascular coupling mechanisms. Chen et al. used their new hybrid fluorescence and optoacoustic tomography (FLOT) system to study the connection between brain hemodynamics and calcium responses to a given sensory stimulation [171]. A fiberscope was inserted through the central channel of a hemispherical PA array probe placed on the top of a scalp-removed mouse skull (Fig. 11a). Five distinct wavelengths (700, 730, 755, 800, and 850 nm) were used for illumination while the mouse was stimulated with a electric burst applied to its left hind limb. Together with spectrally unmixed images of HbO_2_ and HbR, the fluorescence image stack simultaneously acquired under a stimulation pulse train was analyzed following the general linear model-based FLOT processing pipeline. The resulting hemodynamic response function map and GCaMP calcium response function map were superimposed on the mouse’s cerebral ROI. The linearity of the revealed partial PA biomarker increases (normalized peaks of ΔHbO_2_/HbO_2_, ΔHbR/HbR, ΔHbT/HbT, ΔSO_2_/SO_2_) and the partial FL biomarker increase (normalized peak of ΔF/F) showed the coupling between calcium-associated neural activity followed by hemodynamic responses (Fig. 11b, c). Employing the similar approach, Chen et al. established concurrent magnetic resonance optoacoustic tomography (MROT) and revealed the close correlation between functional PACT signals and blood oxygen level-dependent (BOLD) signals from functional magnetic resonance imaging (fMRI) (Fig. 10d) [172]. Interestingly, as seen in Fig. 11e, f, activation intensity, time to peak and maximum t-value (|tmax|), partial HbO_2_ and HbR increase rate exhibited superior sensitivity to that of BOLD signal increase rate toward cerebral blood perfusion responses to external stimulus, proposing MROT as a new modality to enlighten comprehensive investigation of neurovascular coupling mechanism.

**Fig. 12:**
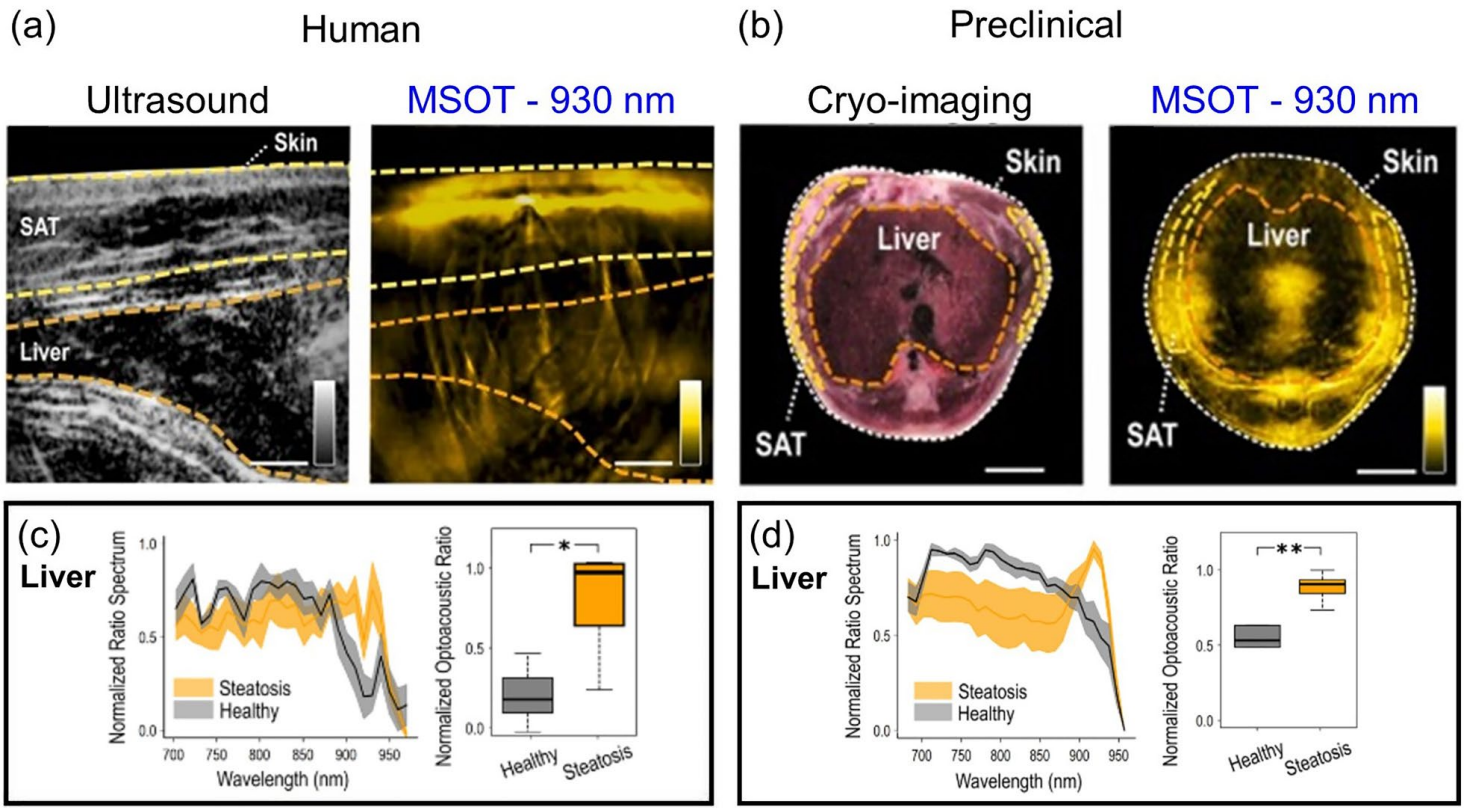
Label-free characterization of intrahepatic fat accumulation in liver steatosis, using near-infrared MSOT. **a** Clinical MSOT images with ultrasound-aided ROI delineation of subcutaneous fat (SAT) and the liver. **b** Preclinical MSOT imaging with cryoimaging-aided ROI selection of SAT and the liver. Comparative normalized ratio spectra, and normalized liver ROI/background mean pixel intensity ratio at 930 nm for **c** for the whole participant cohort (steatotic, n = 5; healthy, n = 5), and **d** for the whole animal model cohort (high fat diet, n = 5; regular chow diet, n = 5). The images are reproduced with permission from [173]

Beyond the hemoglobin-derived biomarkers, broader functional PACT biomarkers can capture more comprehensive physiological changes in organs by targeting different target chromophores, the angiographic morphology, or even hemodynamic flow. If the spectral window with major hemoglobin absorption can be avoided, secondary chromophores—such as melanin, lipid, and collagen—composing a biological tissue can also be selectively targeted. For instance, there is rich absorption by lipid in the spectral region around 930 nm, and this spectral characteristic was exploited by Fasoula et al. to measure lipid accumulation in the livers of patients with hepatic steatosis [173]. Their investigation began with a pilot study involving 5 participants with steatosis and 5 without, and used a clinical hand-held MSOT/US system. Significant observations made during this pilot study were then validated and repeated with preclinical MSOT in a rodent model fed a high-fat diet. Next, 28 (human) or 29 (animal) wavelengths were selected for multispectral imaging in the 680–960 nm range, while the 900–970 nm region was chiefly used for lipid detection. The contours of the liver region, the subcutaneous adipose tissue (SAT) region, and the entire tissue region were carefully traced onto each multispectral stack based on their anatomical position in the co-registered ultrasound images or cryoimaging section (Fig. 12a, b). In the normalized PA spectra of each ROI, compared to the spectra of healthy volunteers, prominent absorption increases at 930 nm were confirmed in steatotic liver patients, while no significant SAT signal rise was identified (Fig. 12c, d). Further, in the liver ROIs of 930 nm monowavelength PA images, the areas under the PA intensity distribution above the intersection point of the two-group histogram were notably higher in the steatotic patient group (*p*-value < 0.001). This finding was highly similar to the result of an identical analysis repeated with accompanied preclinical data, signifying that MSOT can detect and monitor hepatic steatosis noninvasively in clinical circumstances.

**Fig. 13:**
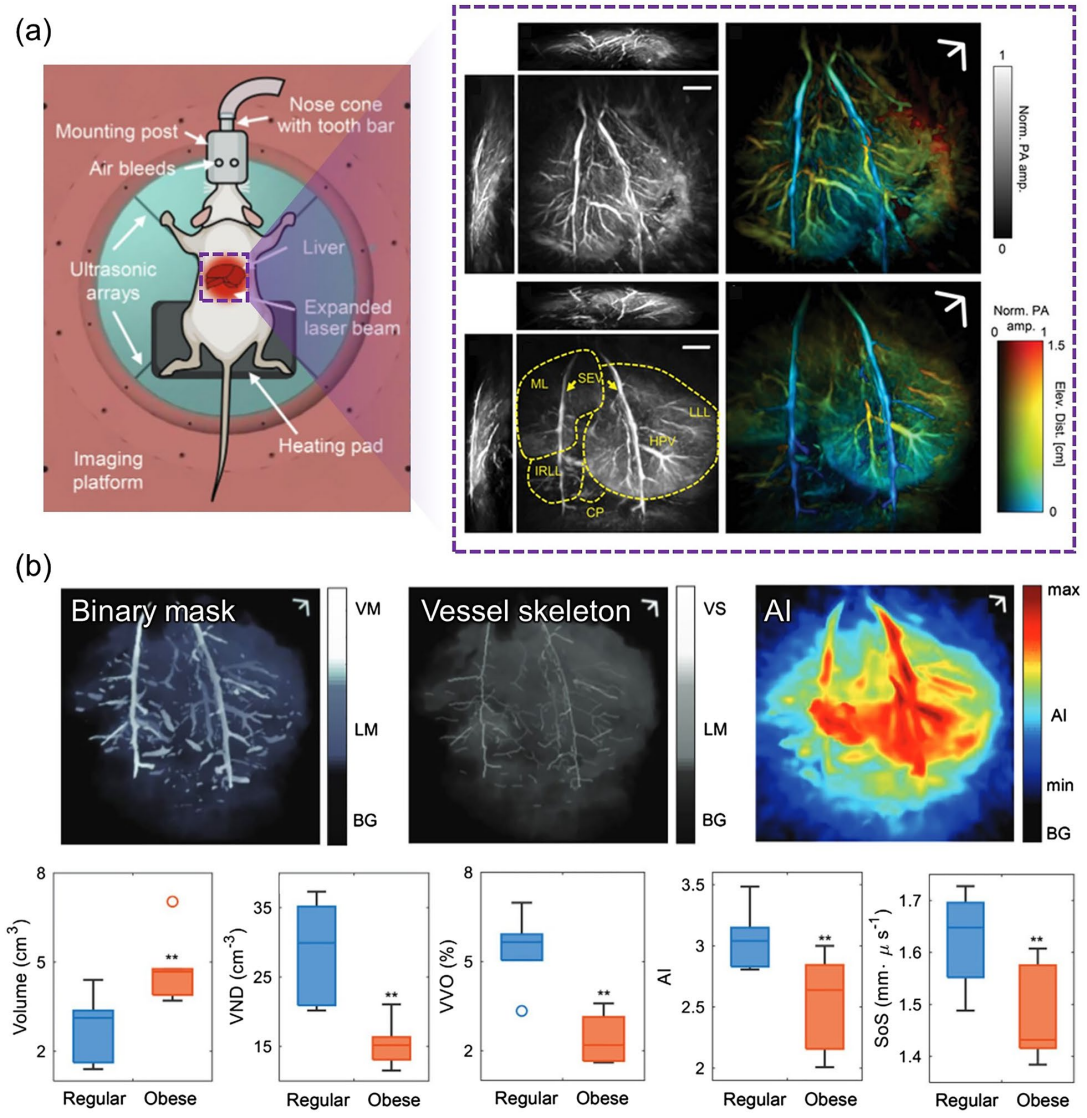
Changes in the angiographic morphology and hemodynamics of rat fatty livers in vivo. **a** Schematic of the liver 3D-PACT setting with an anesthesized animal, and representative 3D PA volume MAPs and depth-encoded angiograms acquired from lean (upper right) and obese (lower right,) rat livers. **b** Quantitative biomarker results between the lean and obese groups. VM, vessel mask; LM, liver mask; BG, background; VS, vessel skeleton; AI, angiographic irregularity; VND vessel number density; VVO: vessel volume occupancy; SoS: speed of sound. The images are reproduced with permission from [84]

Studying a similar target disease, Tong et al. imaged vascular anatomy changes in non-alcoholic fatty liver disease by using 3D PACT with four arc-shaped ultrasonic transduver arrays [84]. Whereas the aforementioned liver steatosis study characterized the photochemical property of lipid via multispectral imaging, this study precisely focused on comparing changes in vessel morphology and acoustic properties from clear volumetric vasculatures imaged at an isotropic spatial resolution of 0.38 mm (Fig. 13a). To achieve the utmost image quality, deformations induced by respiratory motions were cancelled using time-gated reconstruction. After applying 3D high pass filtration and Hessian-based Frangi vessel filtering to enhance the blood contrast, the liver and vessels were segmented into binarized mask and the voxel number between two masks were quantified into vessel volume occupancy. For further morphological analysis, vessel centerlines were extracted by skeletonization process. More than vessel number density, the group devised a new integrative measure termed ‘angiographic irregularity’, which is a product of vessel distribution diversity and morphological irregularity. Finally, the velocity of sound was approximated by taking into account the decreased speed of sound in the lipid-rich liver, which is an essential consideration in the ultrasonographic diagnosis of hepatic conditions. From all five biomarkers, statistically clear distinctions were observed between lean and obese rats (Fig. 13b). All studies reviewed in this section are summarized in Table 4.

**Table 4:** Summary of functional photoacoustic computed tomography systems’ specifications

Ref.	System scale	Functionality and application	Transducer		Laser		Lateral resolution (μm)	Imaging depth (mm)
			f (MHz)	Element	PRF	Wavelength (nm)		
[87]	PACT	Oxygen saturation	2	1024	20	750, 850	380	12.8
[86]	PACT	Cerebral hemodynamics; HbO_2_, HbR, HbT, SO_2_	5.4	512	200	532, 594	170	3.5
[171]	PACT	Cerebral hemodynamics; HbO_2_, HbR, HbT, SO_2_	7	512	20 (100/5)	700, 730, 755, 800, 850	133	4
[172]	PACT	Cerebral sensory response hemodynamics; HbO_2_, HbR, HbT, SO_2_	5	384	10 (50/5)	700, 730, 755, 800, 850	163	4
[173]	PACT	liver steatosis; Normalized ratio spectrum, normalized optoacoustic ratio at 930 nm	5/4	256/256	10/25	680–960 (10 nm step)/700–970 (10 nm step)	300	34
[84]	PACT	liver steatosis; Liver mask volume, vessel number density, vessel volume occupancy, angiographic irregularity, speed of sound	2.25	4 × 256	12.5	1064	380	12
[120]	PACT	Venous flow hemodynamics; flow speed, flow vector map, fractional speed change	13–24	256	10/20/100	1064/670–1070	125	5.5

f, center frequency; PRF, pulse repetition frequency; PACT, photoacoustic computed tomography

## Conclusions

The scalability of fPAI makes it a highly promising technology for a wide range of biomedical applications, spanning from the examination of human organs to imaging individual cells. In addition, its functional imaging capabilities can provide quantifiable data about disease-relevant parameters, including HbT, SO_2_, blood flow velocity, temperature, and MRO_2_. The multi-scalable and multi-functional nature of PAI has been demonstrated through its successful applications in various domains, including high-resolution imaging of brain hemodynamics in small animals, diagnosis of deep-seated cancers, and observation of human brain activity.

This review summarizes recent advances in the field of fPAI, which has shown remarkable results in various biomedical applications. The latest fPAI systems fall into into two categories: (1) fPAI in the acoustic resolution regime has enabled the mapping of lymphatic vessels and whole-body imaging in small animals. The recent development of deep-learning-assisted PACT using a 1024 spherical array transducer has enabled the monitoring of brain hemodynamics and changes in oxygen levels in rats. (2) fPAI in the optical resolution regime has enabled recent studies using high-resolution OR-PAM to evaluate cerebral hemodynamics and angiogenesis around tumors. In particular, many attempts are being made to diagnose stroke and cancer, and the imaging speed, imageable area, and resolution for this purpose have been remarkably improved. Recent studies that simultaneously captured super-resolution PA images and topological properties have contributed greatly to this field, suggesting a new direction for SR-PAM.

Advances in fPAI technologies, particularly those applied to small animal and nanoscale samples, have demonstrated great clinical potential. The development of PACT by Wang et al. has significantly advanced observations of detailed vascular structures in the human breast [66] and of brain activity [174]. The fPAI breast cancer screening developed by Oraevsky et al. has been granted a Premarket Approval Application (PMA) approval by the US Food and Drug Administration (FDA) for commercial distribution in the US [49, 111]. The multispectral optoacoustic tomography (MSOT) developed Ntziachristos et al. is being utilized globally by medical professionals to monitor a range of diseases, including Crohn’s diseases [175], dermatologic diseases such as psoriatic and atopic dermatitis [176, 177], and Duchenne muscular dystrophy [134]. Kim et al. are leading the development of a programmable photoacoustic and ultrasound imaging (PAUSI) system and have applied it to the diagnosis of diseases such as thyroid cancer [62] and melanoma [72], and to foot imaging [76].

The future of fPAI holds immense promise for advancing biomedical research and clinical diagnostics. By focusing on improving imaging depth and resolution, advancing contrast agents and molecular probes, integrating multispectral and multimodal imaging, enabling real-time and dynamic imaging, and facilitating clinical translation and validation, functional PAI can reach new heights in terms of sensitivity, specificity, and clinical relevance. These advancements will pave the way for enhanced disease diagnosis, treatment guidance, and monitoring, ultimately benefiting patients and advancing healthcare.

## Data Availability

Not applicable.
